# Prognostic immunogenic characteristics of iron pendant disease modifiers in colon cancer

**DOI:** 10.3389/fimmu.2023.1100725

**Published:** 2023-05-25

**Authors:** Xian Wang, Qingyu Meng, Yawen Chen, Yanjun Zhang, Xiaohui Huang, Longquan Xiang, Haiyang Kong, Chunxi Wang, Xueyang Wang, Dekang Zhang

**Affiliations:** ^1^ Department of General Surgery, the First Medical Center, Chinese PLA General Hospital, Beijing, China; ^2^ Department of Health Management, The Second Medical Center and National Clinical Research Center for Geriatric Diseases, Chinese PLA General Hospital, Beijing, China; ^3^ Department of Radiology, the First Medical Center, Chinese PLA General Hospital, Beijing, China; ^4^ Department of Graduate ,Medical School of Chinese PLA, Beijing, China; ^5^ Department of Pathology, Jining NO.1 People’s Hospital, Shandong Jining, China; ^6^ Department of General Surgery, Qufu Hospital of Traditional Chinese Medicine, Qufu, China; ^7^ Department of Radiology, Yancheng Traditional Chinese Medicine Hospital Affiliated to Nanjing University of Chinese Medicine, Yancheng, China

**Keywords:** colon cancer, iron pendant disease modifier, prognosis, immunogenic characteristics, prognostic risk model

## Abstract

**Background:**

We explored the prognostic and immunogenic characteristics of iron pendant disease regulators in colon cancer to provide a scientific basis for the prediction of tumor prognosis-related markers and potential immunotherapeutic drug targets.

**Methods:**

RNA sequencing and matched complete clinical information of colon cancer (COAD) were retrieved from the UCSC Xena database, and genomic and transcriptomic data of colon cancer from the TCGA database were downloaded. Then univariate and multifactorial Cox regression were used to process these data. The prognostic factors were analyzed by single-factor and multi-factor Cox regression, followed by Kaplan-Meier survival curves with the aid of R software “survival” package. Then we use FireBrowse online analysis tool to analyze the expression variation of all cancer genes, and draw a histogram according to the influencing factors to predict the 1, 3, and 5 year survival rates of patients.

**Results:**

The results show that age, tumor stage and iron death score were significantly correlated with prognosis (p<0.05). Further multivariate cox regression analysis confirmed that age, tumor stage and iron death score were still significantly correlated with prognosis (p<0.05); The calibration curve results show that the deviation between the predicted values of 1 year, 3 years and 5 years and the diagonal of the figure is very small; the ROC curve results show that the AUC values of the 1-year and 5-year ROC curves of the bar graph are high; the DCA curve results show that the net yield of the bar graph is the largest; The scores of T cells and B cells in the high iron death score group were significantly lower than those in the low iron death score group, and the activities of immune related pathways were significantly reduced. There was a significant difference in the iron death score between the iron death molecular subtype and the gene cluster subtype.

**Conclusions:**

The model showed a superior response to immunotherapy in the high-risk group, revealing a potential relationship between iron death and tumor immunotherapy, which will provide new ideas for the treatment and prognostic assessment of colon cancer patients.

## 1 Introduction

Since the 21st century, the incidence of colorectal cancer in China has remained high (1). 2018 Chinese cancer statistics show that the incidence and mortality rate of colorectal cancer in China occupy two of the top five positions among all malignant tumors, which is a high incidence cancer, and the incidence of colorectal cancer is still on the rise, and the incidence of colorectal cancer in urban population is much higher than the incidence of colorectal cancer in rural population (2, 3). The disease can lead to abdominal pain, abdominal lumps and other symptoms, and in serious cases, metastasis may occur, leading to death, which poses a threat to patients’ physical and psychological health and life safety (4–6). At the same time, colon cancer has the characteristics of high incidence and insidious development, so that in most cases, patients only seek medical treatment when they have symptoms in the middle and late stages. The complexity of the regulatory mechanism of colon cancer can be reflected in the fact that the process of colon cancer development can be regulated in many aspects and levels through multiple signaling pathways (7–10). In addition, colon cancer is influenced by the external environment, which cannot be ignored. It has been found that factors such as alcohol consumption, smoking, genetics, immune deficiency and high fat may be related to the occurrence of colon cancer. Although surgical procedures, radiotherapy, chemotherapy and other treatments have been actively developed, the insidious onset of colorectal cancer leads to the fact that most patients are already in the middle and late stages of the tumor when they are diagnosed. So most of them lose the opportunity of standard treatment, and the prognosis of colorectal cancer patients is poor (11–14), which includes late detection of the disease. Therefore, the existing treatment methods are no longer able to maximize the effect on patients’ prognosis and survival quality. With the development of oncology, immunology, molecular biology and other related disciplines and interdisciplinary cross cutting content, and the continuous optimization of technical means, researchers have been able to further study and understand the impact of tumor microenvironment and gene level on tumors, which has led to the rapid development of tumor immunotherapy research (15, 16). More and more tumor immunotherapy targets have been discovered by researchers one after another. In the context of the development of big data era, immunotherapy has gradually become a new research hotspot and research focus in tumor treatment, and a new direction for colorectal cancer treatment (17). The research and development of immunotherapy have improved the prognosis of some colorectal cancer patients to a certain extent. Research has found that there are multiple prognostic genes in various human cancers. Prognostic gene identification based on genome database is helpful to determine the prognostic impact of cancer and understand the progress of cancer (18). Therefore, it is possible to benefit more patients from scientific research by conducting research related to immunotherapy by starting from colon cancer-specific genes, such as immune-related genes, and thus proceeding along the research path of further improving the treatment methods for colorectal cancer patients. Currently, some progress has been made in the screening of prognostic genes for colon cancer, and previous studies have shown that iron death may be a potential rake point for tumor growth inhibition and immunotherapy (19–22). Based on this, this study combined bioinformatics approach with real-world clinical data, using bioinformatics analysis of gene microarrays and high-throughput sequencing data published in major public databases such as TCGA and GEO, and mining gene expression profile data and transcriptome data published in databases for differential gene analysis between tumor and normal paired samples, so as to provide prediction of tumor prognosis-related markers and potential immunotherapeutic drug targets.

## 2 Methods

### 2.1 Data acquisition and processing

RNA sequencing and matched complete clinical information (age, sex, survival status, tumor stage) for colon cancer (COAD) were retrieved from the UCSC Xena database, and genomic and transcriptomic data for colon cancer from the TCGA database were downloaded. After filtering samples without survival status and survival time, samples with both genomic, transcriptomic and clinical data were retained, resulting in a total of 426 Count values were normalized to the number of transcripts per kilobase million (TPMs).

Combining the literature data and the iron death database, the intersection of related gene sets was taken to obtain a total of 69 iron death suppressor regulators, and 68 of which were present in TCGA-COAD. The analysis was performed by ConsensusClusterPlus package, and principal component analysis (PCA) was used to examine the discrimination between subtypes after clustering. Similarly, consistency clustering based on differential genes among iron death subtypes was performed using the same method for clustering and discriminant testing. We collected the original expression profile data and clinical survival data of the COAD tumor samples GSE39582 and GSE17536 from the GEO database, and used the affy package to process the CONSENSUS clustering data of the TCGA-COAD dataset based on 69 iron death inhibitors. Then use the CONSENSUSClusterPlus package for analysis. After clustering, principal component analysis (PCA) was used to check the differences between subtypes. Similarly, consistency clustering based on differential genes among iron death subtypes was performed using the same method for clustering and discrimination test. Based on the mRNA expression profiles of 68 FRGs in COAD samples from the TCGA database, COAD patients were classified into three molecular patterns (C1:n=81; C2:n=226; C3:n=119) by unsupervised cluster analysis to assess the correlation between these patterns and the characteristics of the tumor immune microenvironment, as well as the biological behavior among the three iron death molecular patterns. The iron death score of each COAD sample was calculated according to the principal component analysis (PCA), and then all patients were divided into high iron death score group and low iron death score group, and their correlation with the prognosis of colorectal cancer was analyzed. At the same time, the age and clinical stage information of patients were collected, and their impact on the prognosis was analyzed using univariate and multivariate Cox regression, respectively. Then the Kaplan Meier survival curve was drawn using R software, FireBrowse online analysis tool was used to analyze the difference of gene expression in different cancer species, and a line graph was constructed based on influencing factors to predict the survival rate of patients in 1, 3 and 5 years. The GSE39582 and GSE17536 datasets were used as validation datasets to validate this prognostic risk model. Finally, PCA and Tsne clustering results were analyzed. Tumor samples were screened for high and low risk as a new grouping basis, and differential gene reanalysis was performed on the training set (TCGA) and validation set (GEO) data.

### 2.2 Statistical analysis

R4.0.3 software and Graphad Prism 9.0 software were used for statistical analysis and graphing. Wilcoxon rank sum test and Kruskal-Wallis test were used for comparison between groups. Kaplan-Meier survival curves were used to analyze the survival of patients with colon cancer. The data obtained in R language were labeled using the mean ± standard deviation (SD). When the obtained data conformed to a normal distribution with a statistically significant two-by-two comparison, the obtained data were subjected to t-test for correlation analysis, and ANOVA test was used for analysis when multiple groups were compared. For data that did not conform to a normal distribution, the Wilcoxon test was used for correlation analysis. The correlation of the clinical data of the patients was argued by the statistical X2 test. The Kaplan-Meier survival curves were then plotted using the R software “survival” package, and the FireBrowse online analysis tool was used to analyze the differences in gene expression across cancer species. Column plots were constructed to predict patient survival at 1, 3, and 5 years, with overall survival defined as the time from the day the patient underwent tumor resection to the day the patient died. The GSE39582 and GSE17536 datasets were used as validation datasets to validate this prognostic risk model, and finally PCA and Tsne clustering results were analyzed. Subject work characteristic (ROC) curves were used to evaluate the efficacy of the prognostic risk model in predicting 1, 3, and 5 year survival of colon cancer patients. The test level α for this study was 0.05, and all data were further analyzed using SPSS 22.0 as well as GraphPad Prism 9.0.2.

## 3 Results

**Figure 1:**
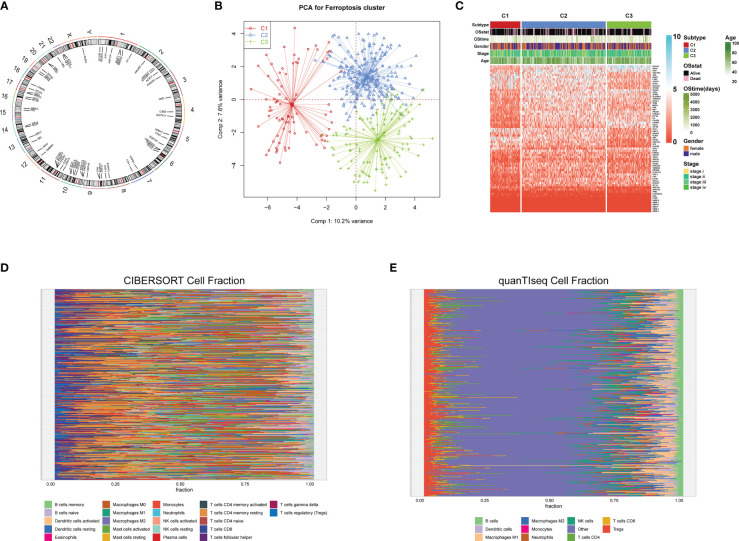
Copy number variation (CNV) locations of these FRGs on the chromosomes**(A)**;Principal component analysis **(B)**;Three patterns of COAD patients with different clinicopathological features**(C)**; The correlation between these patterns and the immune microenvironmental characteristics of the tumors **(D, E)**.

**Figure 2:**
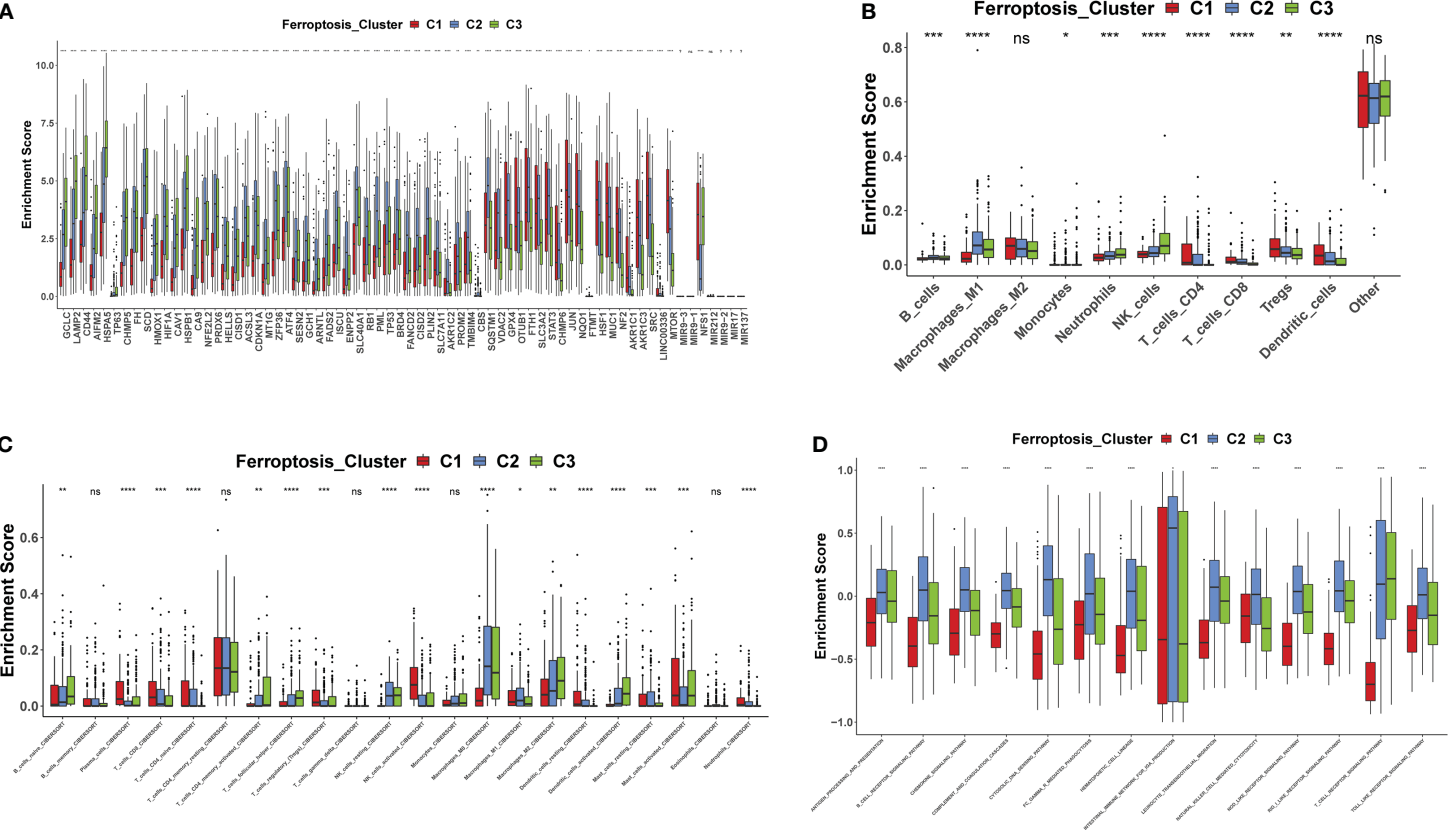
FEGs differences between the three molecular patterns **(A–D)**. *: p<0.05; **:p<0.01; ***: p<0.001; ****:p<0.00001; ns, no significance.

A total of 69 FRGs were included in this study, 68 of which were present in TCGA-COAD. **Figure 1A** shows the copy number variation (CNV) locations of these FRGs on the chromosomes. Based on the mRNA expression profiles of 68 FRGs in COAD samples from The Cancer Genome Atlas (TCGA) database, COAD patients were classified into three molecular patterns (C1:n=81; C2:n=226; C3:n=119) by unsupervised clustering analysis. Principal component analysis (PCA) confirmed that these three subtypes were fully distinguishable (**Figure 1B**). **Figure 1C** shows the three patterns of COAD patients with different clinicopathological features. In addition, we assessed the correlation between these patterns and the immune microenvironmental characteristics of the tumors. Our data showed variability in the degree of immune cell infiltration in different samples (**Figures 1D, E**), significant differences in FEG expression values between almost all three molecular patterns, and regular differences in the expression of iron death inhibitory regulator genes in the three molecular patterns, such as GCLC, CD44, and other genes with the lowest expression in pattern I and the highest expression in pattern III (**Figure 2A**); and SRC, MTOR. There were also significant differences in immune cell infiltration (**Figures 2B, C**) and immune function (**Figure 2D**), especially for B cells and T cells, etc. (*: p<0.05; **:p<0.01; ***:p<0.001; ****:p<0.00001.)

### 3.1 TME characteristics in three ferroptosis gene clusters for COAD

**Figure 3:**
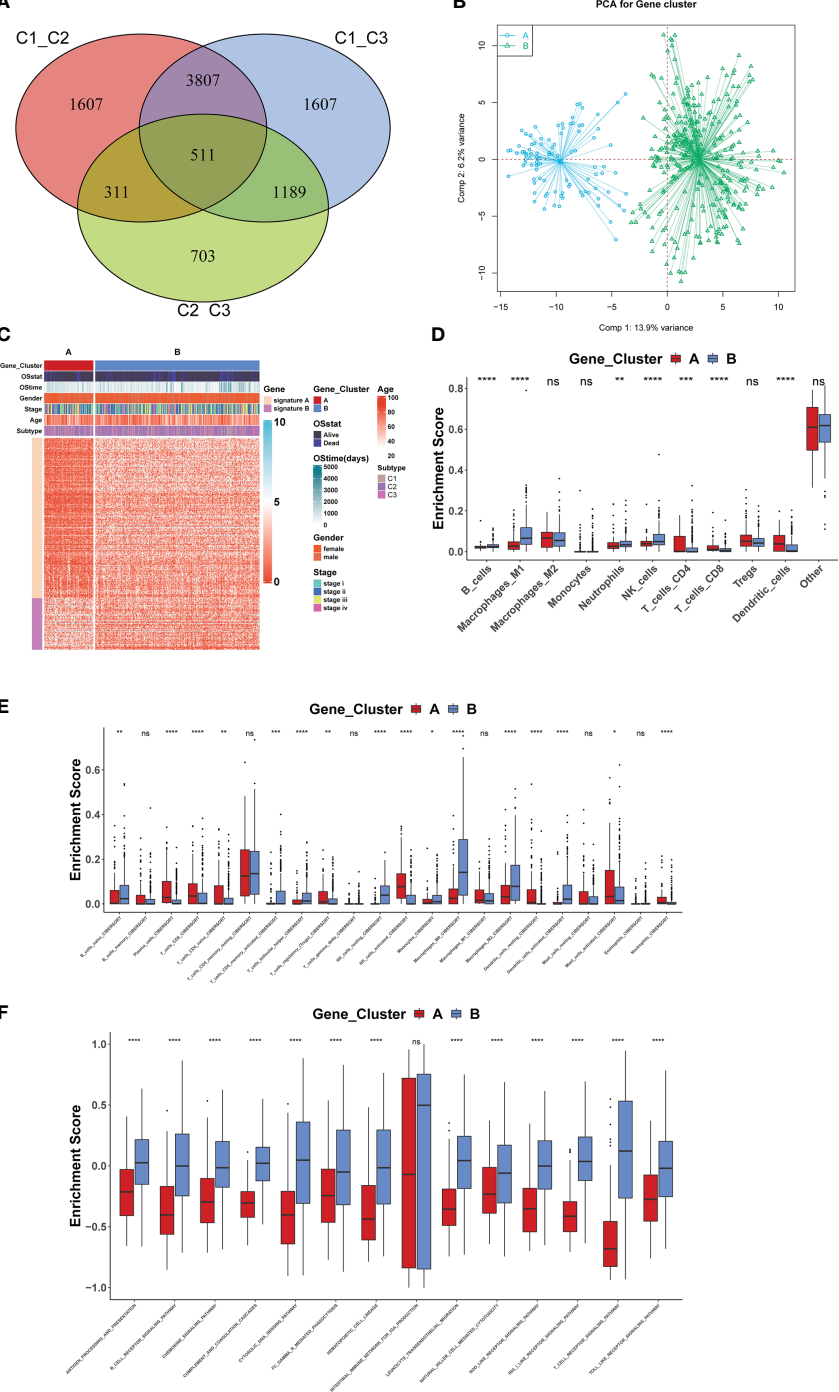
Biological behavior among the three molecular patterns of iron death **(A–F)**. *: p<0.05; **: p<0.01; ***: p<0.001; ****: p<0.00001; ns, no significance.

We further explored the biological behavior among the three molecular patterns of iron death. Differential expression analysis was performed on these three patterns, and the concatenation of the three differentially expressed genes (DEGs) totaling 7425 (**Figure 3A**) was taken for univariate cox regression analysis to screen genes associated with prognosis, resulting in 255 genes. Based on these 255 genes, unsupervised clustering was performed and the TCGA-COAD cohort was divided into 2 gene clusters (**Figure 3B**) as gene cluster A and gene cluster B (**Figure 3C**), respectively. There were significant differences in immune cell infiltration (**Figure 3D, E**) and immune function (**Figure 3F**) between the two gene clusters, especially for B cells and T cells. The 255 genes were classified into gene features A and B using Pearson’s correlation coefficient, where gene feature A represents its positive correlation with gene cluster (r>0), and gene feature B represents its negative correlation with gene cluster (r<0), and they were downscaled using Boruta’s algorithm to obtain 115 gene features A and 31 gene features B.

### 3.2 Development of the ferroptosis scoring system for COAD

**Figure 4:**
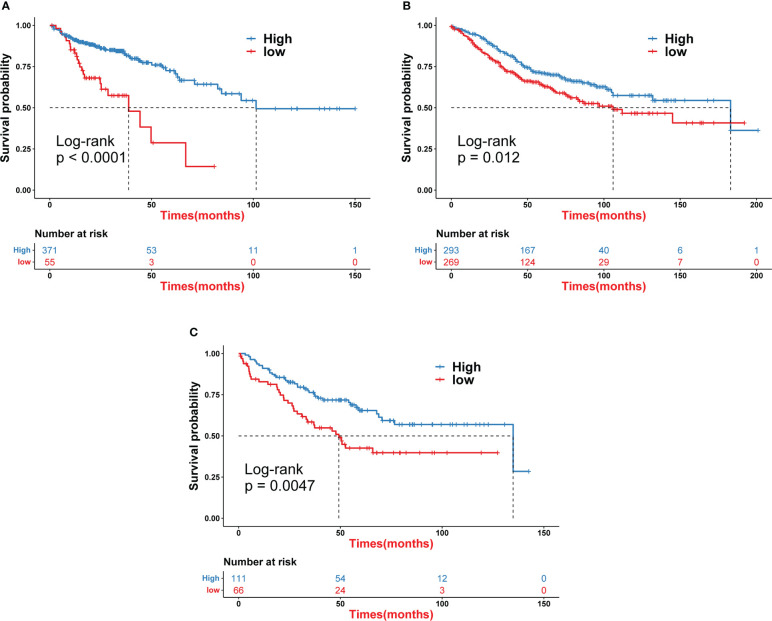
Iron death scores were calculated for each COAD sample based on principal component analysis **(A–C)**.

Iron death scores were calculated for each COAD sample based on principal component analysis (PCA). All patients were then divided into a high iron death score group and a low iron death score group. **Figure 4A** shows the relationship between the high and low iron death score groups of TCGA-COAD samples and prognostic survival. The overall survival was higher in the high iron death score group samples compared to the low iron death score group. Meanwhile, in the two external COAD validation sets, GSE39582 (**Figure 4B**) and GSE175362 (**Figure 4C**), the survival difference between the high and low iron death score groups remained significant (p< 0.05), and both the survival rates of the high score group were greater than those of the low score group, indicating that our iron death score system constructed in TCGA-COAD has good generalizability and strong robustness.

### 3.3 Ferroptosis score as an independent prognostic factor for COAD

**Figure 5:**
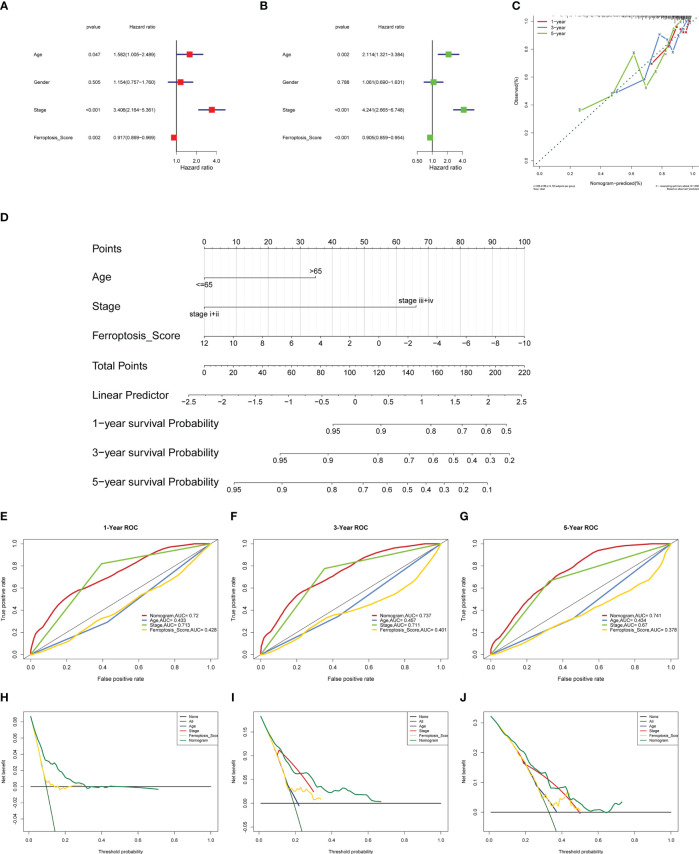
To perform an in-depth analysis of the prognostic value of the iron death score **(A–J)**.

To perform an in-depth analysis of the prognostic value of the iron death score, we performed prognostic analysis of iron death score with other clinical characteristics in the TCGA-COAD cohort, and the results of one-way cox regression analysis showed (**Figure 5A**) that age, tumor stage, and iron death score were significantly associated with prognosis (p< 0.05). Further multifactorial cox regression analysis confirmed (**Figure 5B**) that age, tumor stage, and iron death score were still significantly associated with prognosis (p< 0.05). Therefore, we constructed column line plots (**Figure 5C**) to predict patient survival at 1, 3 and 5 years based on these three clinical characteristics, and validated the accuracy of column line plots to predict prognosis by calibration curves, ROC curves, and DCA curves. The calibration curve results showed (**Figure 5C**) that the predicted values at 1, 3 and 5 years deviated very little from the diagonal of the graph. The ROC curve results showed (**Figure 5E–G**) that the AUC values of the 1, 3 and 5 year ROC curves of the column line graph were high (greater than 0.7), and the DCA curve results found that (**Figures 5H–J**) the net benefit of the column line graph was the largest (compared with other individual clinical features). All three of the above methods all indicate that the column line graph has good accuracy in predicting patient prognosis.

### 3.4 Ferroptosis score is associated with TME features of COAD

**Figure 6:**
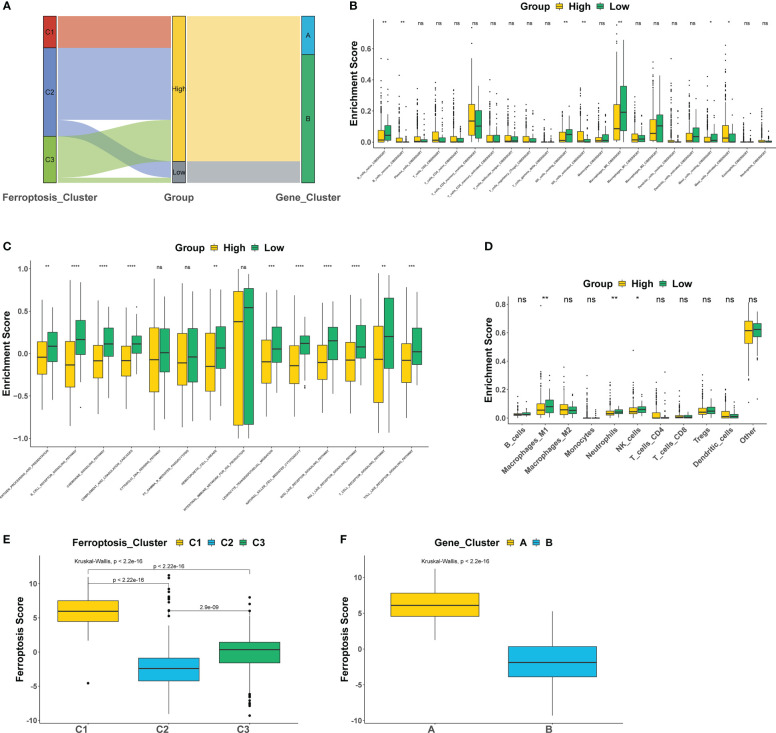
The correlation between iron death scores and TME characteristics **(A–F)**. *: p<0.05; **: p<0.01; ***: p<0.001; ****: p<0.00001; ns, no significance.

This study further explored the correlation between iron death scores and TME characteristics. The data showed that the high iron death score group had significantly lower scores of T cells and B cells (**Figures 6A, B, D**) and lower activity in immune-related pathways (**Figure 6C**) compared to the low iron death score group. Also, the iron death scores differed significantly between iron death molecular subtypes and gene cluster subtypes (**Figure 6E, F**). Taken together, iron death scores were closely associated with TME in COAD.

### 3.5 Characteristics in groups with high or low ferroptosis score

**Figure 7:**
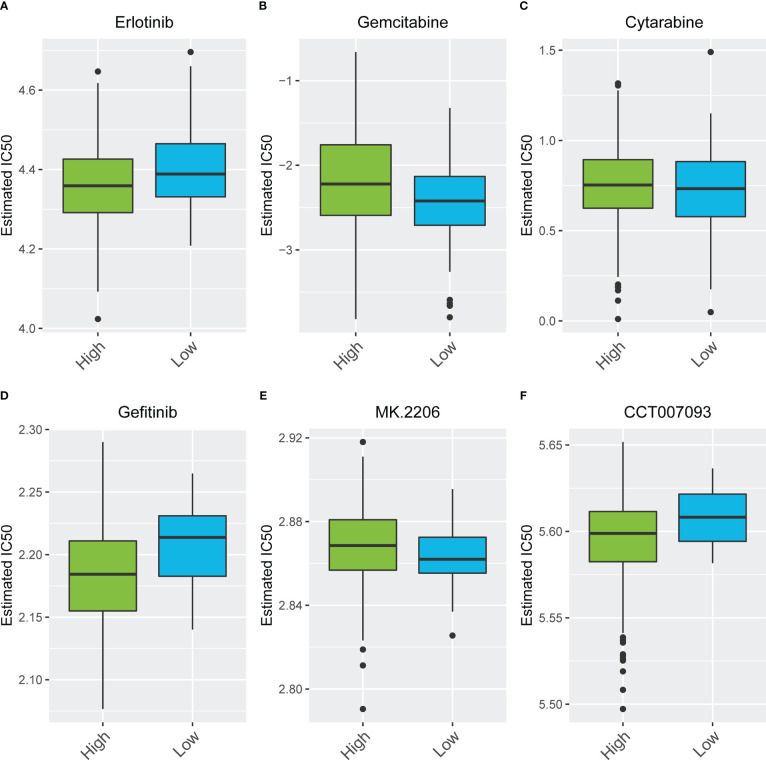
The differences in estimated half-maximal inhibitory concentration (IC50) levels of six chemotherapeutic agents **(A–F)**.

**Figure 8:**
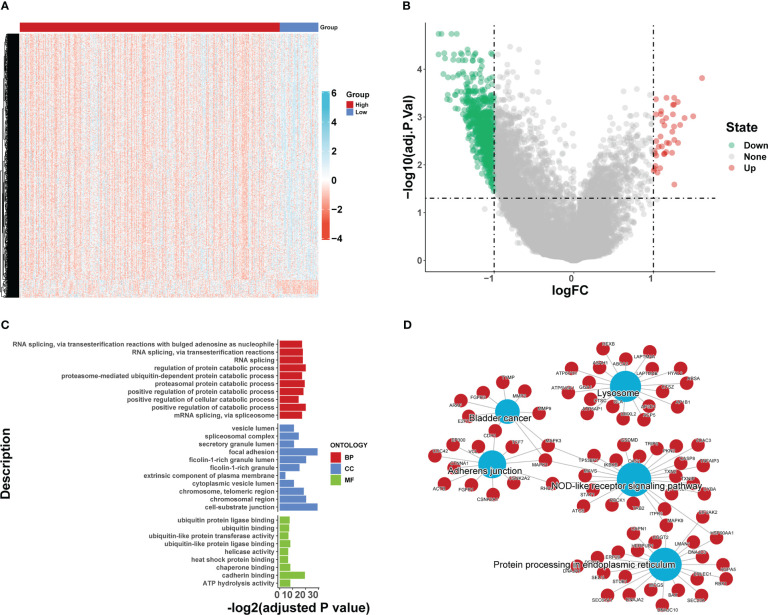
The immunotherapy response in the high and low scoring groups was further investigated based on the GSE78220 and IMvigor210 datasets **(A–D)**.

**Figure 9:**
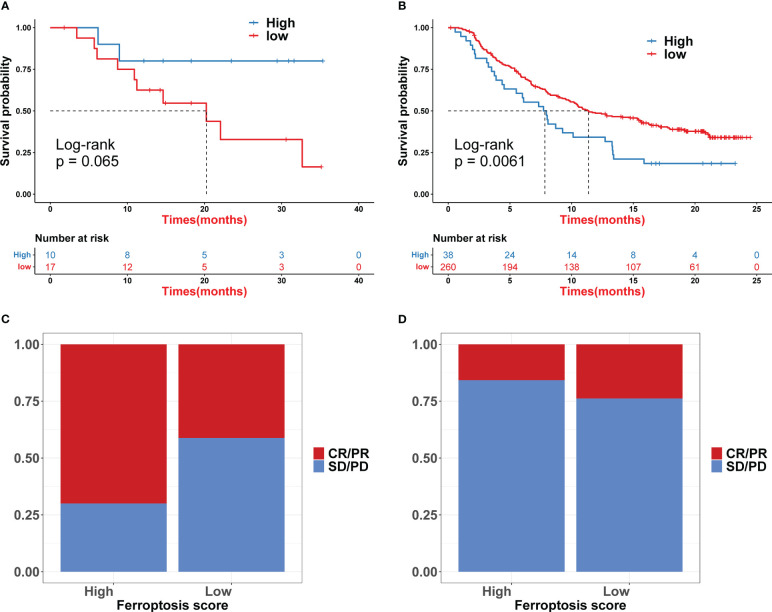
Differential analysis between high and low iron death groups (low vs. high) **(A, B)**, and the results of GO **(C)** and KEGG **(D)** enrichment analysis.

We compared the differences in estimated half-maximal inhibitory concentration (IC50) levels of six chemotherapeutic agents, including erlotinib (**Figure 7A**), gemcitabine (**Figure 7B**), cytarabine (**Figure 7C**), gefitinib (**Figure 7D**), the Akt1/2/3 inhibitor MK.2206 (**Figure 7E**), and the PPM1D (WIP1) inhibitor CCT007093 (**Figure 7F**). Our data showed that the high iron death score group was more sensitive to erlotinib, gefitinib, and PPM1D (WIP1) inhibitor CCT007093 compared to the low iron death score group. In contrast, the low-iron death scoring group was more sensitive to gemcitabine, cytarabine and Akt1/2/3 inhibitor MK.2206. In addition to this, the immunotherapy response in the high and low scoring groups was further investigated based on the GSE78220 and IMvigor210 datasets, and we found that survival in the high and low scoring groups had opposite results in GSE78220 (**Figure 8A**) and IMvigor210 (**Figure 8B**), however, this did not affect the relationship between sample survival and immunotherapy response (**Figure 8C, D**), i.e., the higher the survival rate of the samples, the worse their response to immunotherapy. Finally, we performed differential analysis between high and low iron death groups (low vs. high) to obtain 748 DEGs (upregulated = 40, downregulated = 708) (**Figure 9A, B**), and the results of GO (**Figure 9C**) and KEGG (**Figure 9D**) enrichment analysis showed that these genes were mainly enriched in GO pathways such as RNA splicing, chromosomal regions and protein activation as well as NOD-like receptor signaling pathways, endoplasmic reticulum KEGG pathway such as protein processing.

## 4 Discussion

Colon cancer is one of the most common but preventable malignant tumors of the gastrointestinal tract, mainly caused by the deterioration of benign lesions of the colonic mucosal epithelium, and its incidence is only after malignant tumors of the stomach and esophagus (8, 23). It is the third most common cancer in the world and the second most common malignant tumor in China and the second most common cause of cancer death. Every year, the number of patients dying from colon cancer is increasing. Colon cancer is characterized by high incidence, susceptibility to poor prognosis, genetic and environmental factors (24). The main cause of death of colon cancer patients is due to its insidious onset, slow progression, lack of characteristic clinical manifestations, metastasis in the early stages of tumor lymph nodes and poor prognosis, which is also the reason why the clinical diagnosis is easily confused with diseases such as intestinal inflammatory diseases and functional disorders of the intestine. Studies have found that colon cancer is related to alcohol consumption, smoking, genetics, immunodeficiency, high fat and other factors. With the increasing per capita standard of living in China, changes in eating habits and diet structure, as well as the lack of exercise and excessive mental stress among urban workers, coupled with the increased intake of meat and protein, in addition, the amount of alcohol consumption in life has increased, which makes the average age of onset of patients suffering from colon cancer faster than in Europe and the United States, and the age has gradually become younger (25). The 2014 World Health Cancer Report shows that due to Environmental pollution tends to change heavily from day to day, which may directly or indirectly lead to an increase in the prevalence of malignant tumors and an increase in the number of patients dying from cancer. Environmental pollution in China has been worsening day by day in the past decade, and environmental pollution in cities is worse than in rural areas, which may be part of the reasons for the increased incidence of colon cancer in China, the younger and urbanized incidence of colon cancer, and the lower incidence in rural areas compared to urban areas. Therefore, there are many factors influencing the prognostic risk of colon cancer patients in the clinical setting, including the patient’s age, daily habits, site of tumor occurrence, biological characteristics and tumor stage. The incidence of colon cancer in our country has tended to increase rapidly in the last few years (26–29). Due to the poor compliance of colonoscopy, many patients do not receive physical examination and other preventions before the appearance of typical symptoms, so that the best time for surgery and treatment is lost, thus leading to poor prognosis of colon cancer patients after clear diagnosis. Although the present surgery, radiotherapy and targeted therapy have a certain degree of relationship to prolong the survival time of colon cancer patients, and the multiple treatments carried out at the same time can prolong the survival time of advanced patients, the tumor has a high probability of local recurrence and metastasis after the tumor is diagnosed and treated, the 5-year survival rate of colon cancer patients after surgery is 50%, and 5-20% of colon cancer patients recur after treatment (30–33). Recurrence and metastasis are the leading causes of death from colorectal cancer and a major obstacle for improving the overall survival of colon cancer patients. However, there is no clear standard for the pathogenesis of colon cancer, therefore, early screening is particularly important for clinical diagnosis and treatment assessment of colon cancer and prognosis (34). In order to further improve the clinical treatment of colon cancer patients, it is necessary to continuously research and explore more convenient and rapid screening indicators that are readily accepted by patients, which are important in improving the survival rate after surgery, as well as in assessing the immediate and long-term survival of colon cancer patients (35).

Iron death is a newly discovered mode of programmed tumor cell death in 2012, which can lead to multiple death pathways (e.g., apoptosis, autophagy, scorch death, etc.) and can cause significant differences in the morphology, genetics, and mechanisms of action of cancer cells from other cell death processes, representing a new regulatory pathway for antitumor therapy (36). With the increasing understanding and awareness of iron death in recent years, the complex biological and clinical features of iron death have been analyzed and studied. For example, the pattern of cell death due to iron death is morphologically very different from other cell death pathways, and the morphological features of cells can be characterized by a decrease in intracellular mitochondrial volume, a decrease or disappearance of mitochondrial folds, and an increase in bilayer density following the massive accumulation of iron ions in iron death and its action on tumor cells (37, 38). Iron death has been reported to be genetically regulated by a variety of genes, mainly involving genetic changes in cells and disturbances in human iron homeostasis and lipid metabolism [z In terms of the biochemical reactions and mechanisms occurring within cells, iron death is also very different from other programmed modes of death, mainly: reduced activity of glutathione peroxidase 4 (GPX4), causing a decrease in intracellular glutathione (GSH) and even depletion of This leads to the inability of lipid oxidation to activate the glutathione redox reaction catalyzed by GPX4, which eventually disrupts the redox reaction metabolism while generating large amounts of reactive oxides (ROS) through the Fenton reaction (Fenton), and finally leads to cell death (17, 39, 40). Iron death has excellent antitumor efficacy and great potential in precision medicine. However, the specific key mechanisms of increased iron ions in tumorigenesis, progression, invasion, and metastasis have not been elucidated.

Immunotherapy against tumors is one of the major emerging therapeutic modalities in recent years, and it has been found that immune escape of tumor cells is involved in the processes related to the occurrence, development, metastasis, recurrence, and drug resistance in the treatment of tumor tissues in the human body. The immune escape of tumor cells is one of the main reasons for the low efficacy or even failure of comprehensive immunotherapy (41, 42). Therefore, restoring or reversing the ability of immune cells to recognize and kill tumor cells is the main research problem of tumor immunotherapy. In existing studies and clinical trials, immunotherapy has been shown to have good response effects and long-lasting responses in various solid tumors, such as melanoma, non-small cell lung cancer, and kidney cancer, and immunotherapy for tumors can significantly inhibit tumor progression at different stages and improve the long-term prognosis of patients. In tumor immunotherapy, iron death, a novel cell-regulated death modality, has shown great potential to induce not only tumor-specific immune responses but also to repolarize macrophages from an immunosuppressive M2 phenotype to an anti-tumor M1 phenotype (43). In tumor cells, T cells alter the tumor microenvironment and cause reduced or even depleted levels of cystine and cysteine, key fuels for tumor cells, causing impaired metabolism and ultimately cell death, which can be inhibited or even reversed by inhibiting iron death-related pathways, i.e., when immunotherapy is combined with iron death sensitizer treatment, the effect on tumor progression is significantly better than with iron death alone immunotherapy or iron death sensitizers alone. The anti-tumor mechanism of cytotoxic T cells, i.e., CD8+ T cells, is mainly through the release of perforin and granzyme to specifically identify and kill human tumor cells, while some studies have shown that CD8+ T cells can act on tumor cells through the Fas-fasL pathway to cause apoptosis without harming normal tissue cells. Recent research has shown that the nitric oxide pathway inhibits the role of macrophages in iron death, providing a new opportunity to use tumor cell iron death to modulate intrinsic immunity in human tumors. In nanobased cancer vaccine immunotherapy, iron death can be involved in suppressing the primary tumor and its distant metastasis and improving the efficiency of drug delivery. Although immunotherapy has achieved great success in precision cancer therapy, and tumor progression can be significantly inhibited and even down-staged in immune-responsive patients, only about 30% of tumor patients currently respond to immunotherapy, which leads to this effective therapeutic approach not achieving its goal. However, only about 30% of tumor patients currently respond to immunotherapy, which results in this effective treatment method not achieving its desired effect (44, 45).A linear risk-prognosis model of iron death-related genes will be constructed by bioinformatics analysis and combined with public databases, and the model will be validated by using the data set in the GEO database. Finally, a risk-prognosis model consisting of 6 iron death-related genes will be derived, and the relationship between the iron death risk-prognosis model on tumor immune infiltration, immune transport pathway, and immune efficacy will be analyzed, aiming to The aim of this study is to screen high-risk tumor patients and treat them with effective and precise immunotherapy to maximize the efficacy of immunotherapy.

The results of this study showed that 69 FRGs were included in this study, 68 of which were present in TCGA-COAD. Based on the mRNA expression profiles of 68 FRGs in COAD samples from The Cancer Genome Atlas (TCGA) database, COAD patients were classified into three molecular patterns by unsupervised cluster analysis (C1: n = 81; C2: n = 226; C3:n = 119), and PCA confirmed that the three subtypes were completely distinguishable; there was variability in the degree of immune cell infiltration in different samples, and the expression values of FEG were almost all significantly different among the three molecular patterns; there were regular differences in the expression of iron death inhibitory regulator genes in the three molecular patterns, such as GCLC, CD44, and other genes were least expressed in pattern I and most expressed in pattern III; while SRC and MTOR were the opposite. There were also significant differences in immune cell infiltration and immune function, especially for B cells and T cells; one-way cox regression analysis was performed to screen genes associated with prognosis, and 255 genes were finally obtained. Based on these 255 genes, unsupervised clustering was performed, and the TCGA-COAD cohort was divided into 2 gene clusters, named Gene Cluster A and Gene Cluster B. Immune cell infiltration and immune function were significantly different between the two gene clusters, especially for B cells and T cells. The 255 genes were classified into gene features A and B using Pearson’s correlation coefficient, where gene feature A represents its positive correlation with gene cluster (r> 0), and gene feature B represents its negative correlation with gene cluster (r 0), and downscaled using the Boruta algorithm to obtain 115 gene features A and 31 gene features B. The high-iron death scoring group samples had higher overall survival rates compared with the low-iron death scoring group, while the survival differences between the high and low iron death scoring groups remained significant in the two external COAD validation sets GSE39582 and GSE175362 (p< 0.05) and were both greater in the high scoring group than in the low scoring group, indicating that the iron death scoring system we constructed in TCGA-COAD has good The results of one-way cox regression analysis showed that age, tumor stage, and iron death score were all significantly associated with prognosis (p< 0.05). Further multifactorial cox regression analysis confirmed that age, tumor stage and iron death score were still significantly associated with prognosis (p< 0.05); the calibration curve results showed (**Figure 5C**) that the predicted values at 1, 3 and 5 years deviated little from the diagonal line in the figure; and the ROC curve results indicated that the AUC values of the ROC curves at 1, 3 and 5 years were higher (> 0.7) for the column line plots. The DCA curve results found that the net yield of the column line graph was the largest (compared to other individual clinical features), and all three tests above indicated that the column line graph had better accuracy in predicting patient prognosis; the high iron death score group had significantly lower scores for T cells and B cells (**Figure 6B, D**) and lower activity in immune-related pathways (**Figure 6C**) compared to the low iron death score group, while the iron death scores differed significantly between iron death molecular subtypes and gene cluster subtypes, and the above results can indicate that iron death scores are closely related to TME in colon cancer. Compared with the low iron death score group, the high iron death score group was more sensitive to erlotinib, gefitinib, and PPM1D (WIP1) inhibitor CCT007093. In contrast, the low-iron death scoring group was more sensitive to gemcitabine, cytarabine, and Akt1/2/3 inhibitor MK.2206. In addition to this, the immunotherapy response of high and low-scoring groups was further investigated based on the GSE78220 and IMvigor210 datasets, and we found that the survival of high and low-scoring groups had opposite results in GSE78220 and IMvigor210. However, this did not affect the relationship between sample survival and immunotherapy response, i.e., the higher the survival of the sample, the worse their response to immunotherapy. Finally, we performed differential analysis between high and low iron death groups to obtain 748 DEGs (upregulated = 40, downregulated = 708), and the results of GO and KEGG enrichment analysis showed that these genes were mainly enriched in GO pathways such as RNA splicing, chromosomal regions, and protein activation, and KEGG pathways such as NOD-like receptor signaling pathway and protein processing of endoplasmic reticulum.

After reviewing the relevant literature, Wang Yuqing et al. (46) successfully constructed a prognostic risk model for colon cancer based on iron death-related lncRNAs and established a column line graph that could be used to determine the overall survival rate of colon cancer patients. ITGB1-DT may promote the development of colon cancer, and Zhang Tao et al. (47) showed that iron death-related lncRNAs may play an important role in the tumor immunity of colon cancer patients, which could be used for prognostic analysis of colon cancer patients. All the above findings are close to the present study. P53RRA increased the concentration of intracellular iron and lipid reactive oxygen species while enhancing the growth inhibition induced by Erastin, an iron-induced cell death activator, a mechanism closely associated with iron death in tumors (48–51). In addition, the lncRNA APCDC1L-AS can induce resistance to erktinib (EGFR-tyrosine kinase inhibitor) in lung adenocarcinoma by inhibiting autophagic degradation of epithelial growth factor receptor (EGFR) (52, 53).

This study is innovative because there are few studies on iron death in colon cancer, and even fewer studies combining immune cell infiltration and immune pathways. In this experiment, we used bioinformatics to analyze the expression of iron death differential genes in colon cancer, constructed a linear risk prediction model, and used the model to detect and verify the relationship between iron death-related genes in colon cancer and immune infiltration, as well as to classify patients into high and low risk based on the model, and to investigate the response of each group of patients to immunotherapy, which is somewhat novel and necessary.

This study is innovative because there are few studies on iron death in colon cancer, and even fewer studies combining immune cell infiltration and immune pathways. In this experiment, we used bioinformatics to analyze the expression of iron death differential genes in colon cancer, constructed a linear risk prediction model, and used the model to detect and verify the relationship between iron death-related genes in colon cancer and immune infiltration, as well as to classify patients into high and low risk based on the model, and to investigate the response of each group of patients to immunotherapy, which is somewhat novel and necessary.

## Data availability statement

The datasets presented in this study can be found in online repositories. GSE39582 and GSE17536 from the GEO database.

## Author contributions

(I) Conception and design: XiW and YWC. (II) Administrative support: CXW QYM and DKZ. (III) Provision of study materials or patients:YJZ, XiW and YWC. (IV) Collection and assembly of data: XYW, YWC XHH and QYM. (V) Data analysis and interpretation: XiW, LQX and HYK.(VI) All authors contributed to the article and approved thesubmitted version.
